# PD‐L1 overexpression correlates with *JAK2*‐V617F mutational burden and is associated with 9p uniparental disomy in myeloproliferative neoplasms

**DOI:** 10.1002/ajh.26461

**Published:** 2022-01-21

**Authors:** Jelena D. Milosevic Feenstra, Roland Jäger, Fiorella Schischlik, Daniel Ivanov, Gregor Eisenwort, Elisa Rumi, Michael Schuster, Bettina Gisslinger, Sigrid Machherndl‐Spandl, Peter Bettelheim, Maria‐Theresa Krauth, Felix Keil, Christoph Bock, Mario Cazzola, Heinz Gisslinger, Robert Kralovics, Peter Valent

**Affiliations:** ^1^ Ludwig Boltzmann Institute for Hematology and Oncology Medical University of Vienna Vienna Austria; ^2^ Department of Laboratory Medicine Medical University of Vienna Vienna Austria; ^3^ Cancer Data Science Laboratory, Center for Cancer Research National Cancer Institute Bethesda Maryland USA; ^4^ Department of Internal Medicine I, Division of Hematology and Hemostaseology Medical University of Vienna Vienna Austria; ^5^ Department of Molecular Medicine University of Pavia Pavia Italy; ^6^ Division of Hematology Fondazione IRCCS Policlinico San Matteo Pavia Italy; ^7^ CeMM Research Center for Molecular Medicine of the Austrian Academy of Sciences Vienna Austria; ^8^ Department of Haematology, Internal Oncology and Stem Cell Transplantation Ordensklinikum Linz Elisabethinen Hospital Linz Austria; ^9^ 3rd Medical Department, Hematology & Oncology, Hanuschkrankenhaus Vienna Austria; ^10^ Institute of Artificial Intelligence, Center for Medical Statistics, Informatics, and Intelligent Systems Medical University of Vienna Vienna Austria

## Abstract

Myeloproliferative neoplasms (MPN) are chronic stem cell disorders characterized by enhanced proliferation of myeloid cells, immune deregulation, and drug resistance. *JAK2* somatic mutations drive the disease in 50–60% and *CALR* mutations in 25–30% of cases. Published data suggest that *JAK2*‐V617F‐mutated MPN cells express the resistance‐related checkpoint PD‐L1. By applying RNA‐sequencing on granulocytes of 113 MPN patients, we demonstrate that PD‐L1 expression is highest among polycythemia vera patients and that PD‐L1 expression correlates with *JAK2*‐V617F mutational burden (*R* = 0.52; *p* < .0001). Single nucleotide polymorphism (SNP) arrays showed that chromosome 9p uniparental disomy (UPD) covers both *PD‐L1* and *JAK2* in all MPN patients examined. MPN cells in *JAK2*‐V617F‐positive patients expressed higher levels of PD‐L1 if 9p UPD was present compared to when it was absent (*p* < .0001). Moreover, haplotype‐based association analyses provided evidence for germline genetic factors at *PD‐L1* locus contributing to MPN susceptibility independently of the previously described GGCC risk haplotype. We also found that PD‐L1 is highly expressed on putative CD34^+^CD38^−^ disease‐initiating neoplastic stem cells (NSC) in both *JAK2* and *CALR*‐mutated MPN. PD‐L1 overexpression decreased upon exposure to JAK2 blockers and BRD4‐targeting agents, suggesting a role for JAK2‐STAT5‐signaling and BRD4 in PD‐L1 expression. Whether targeting of PD‐L1 can overcome NSC resistance in MPN remains to be elucidated in forthcoming studies.

## 1 INTRODUCTION

Myeloproliferative neoplasms (MPN) are chronic bone marrow (BM) disorders characterized by clonal hematopoiesis, overproduction of myeloid cells, inflammation, and immune deregulation.^1^, ^2^ The three classical *BCR‐ABL1*‐negative MPN are essential thrombocythemia (ET), polycythemia vera (PV), and primary myelofibrosis (PMF). The *JAK2*‐V617F mutation drives the disease in ~95% of PV patients and 50%–60% of all cases with ET and PMF.^3^, ^4^, ^5^, ^6^ Somatic mutations affecting *CALR* are disease drivers in 30%–40% of ET and PMF patients,^7^ while *MPL* mutations account for 5%–10% of all MPN cases.^8^, ^9^ MPN patients with unknown disease drivers (no driver mutation detected) are referred to as ´triple negative´ MPN. Progression to secondary acute myeloid leukemia (sAML) may occur in all three MPN entities, but is more frequently documented in patients with PMF compared to those with ET and PV.^10^ Most of the current therapeutic approaches in MPN are not curative, and disease management focuses on amelioration of symptoms.^11^, ^12^ The only exception is allogeneic hematopoietic stem cell transplantation, a procedure that is associated with a relatively high mortality risk, and is, therefore, performed only in younger patients with advanced disease or sAML.^11^ Lack of curative potential and commonly occurring resistance to drug therapies applied in MPN patients highlight the need for the development of novel therapeutic concepts. One strategy may be to establish drug‐based therapies or antibody‐based immunotherapies capable of targeting and eradicating the disease‐initiating neoplastic stem cells (NSC) in MPN.^13^

While immune dysregulation in MPN has been well documented, the mechanisms by which the MPN (stem) cells escape the antitumor immune response remain unclear.^1^ Published studies have shown that MPN cells display certain immune checkpoint molecules, including PD‐L1, which may contribute to resistance, and that constitutive activation of JAK2 by the V617F mutation causes PD‐L1 upregulation in MPN cells.^14^, ^15^

PD‐L1 and PD‐L2 expression by neoplastic cells and binding of these molecules to the PD‐1 receptor on T cells inhibits the antigen‐specific Tcell‐mediated antitumor immune response, and represents a major mechanism of immune escape.^16^, ^17^ PD‐L1 is not only expressed on cells of many tumors, but also cells within the tumor microenvironment in inflammatory conditions, thus reducing antitumor immunity.^18^, ^19^ Upregulation of PD‐L1 in cancer cells can be a consequence of *PD‐L1* gene amplification, activation of oncogenic signaling pathways, or epigenetic regulation, but it can also be induced by certain pro‐inflammatory cytokines.^20^ Blocking of the interaction between the PD‐1 receptor and its two ligands potentiates antitumor T‐cell responses and in line with this notion, checkpoint inhibitors targeting the PD‐1/PD‐L1 interaction have emerged as highly promising anticancer drugs, leading to durable responses in various solid tumors.^21^, ^22^, ^23^, ^24^ The use of PD‐1/PD‐L1 interaction‐targeting agents in MPN is currently under investigation, however, it is unclear, which MPN subtypes are most suitable for testing in clinical trials.

The genes encoding the PD‐L1 (*CD274*, alias *PD‐L1*), PD‐L2 (*CD273*, alias *PD‐L2*), and JAK2 proteins are located in close proximity on the short arm of chromosome 9, which is often affected by copy number neutral loss of heterozygosity through the mechanism of acquired uniparental disomy (UPD) in MPN patients, usually leading to an increase in the *JAK2*‐V617F mutational burden.^25^ UPD of chromosome 9p is the most common chromosomal aberration found in MPN. It affects up to 80% of PV patients, close to half of PMF and sAML patients, and 6%–18% of cases with ET.^26^ However, it is unknown whether chromosome 9p UPD affects *PD‐L1/2* genes, which have a more centromeric position than *JAK2*. In particular, it is not known whether chromosome 9p UPD affects *PD‐L1/2* expression levels in MPN cells.

In the current study, we analyzed *PD‐L1* expression in neoplastic cells in a well‐characterized cohort of MPN patients, for which in‐depth genomic data were available. We combined whole‐transcriptome data with chromosomal aberration profiles based on SNP arrays and mutational profiles from targeted sequencing using a myeloid gene panel. We show that *PD‐L1* overexpression in MPN is associated with chromosome 9p UPD and that it correlates with *JAK2*‐V617F mutational burden in granulocytes. We also found that germline genetic factors at the *PD‐L1* gene locus contribute to MPN susceptibility, and we demonstrate that PD‐L1 is upregulated on the cell surface of phenotypically defined MPN NSC.

## 2 METHODS

### 2.1 Patients and primary cell isolation

Samples from 181 MPN patients and 14 healthy donors were collected at the Medical University of Vienna and Hanusch Hospital in Vienna, Austria; Elisabethinen Hospital in Linz, Austria; and Fondazione IRCCS Policlinico San Matteo in Pavia, Italy. All patients and healthy donors provided informed consent in accordance with the Declaration of Helsinki. The study was approved by local ethics committees. Patients´ characteristics are shown in Table S1. Diagnostic criteria were applied as described previously.^5^ Peripheral blood (PB) samples were collected from healthy donors and MPN patients and BM samples from MPN patients during routine investigations. Granulocytes and mononuclear cells (MNC) were isolated from PB and BM samples according to standard procedures using density gradient centrifugation.

### 2.2 DNA and RNA isolation and driver mutation analysis

DNA and RNA were isolated from granulocyte fractions of PB samples of MPN patients or healthy donors using standard procedures.^5^ *JAK2*, *CALR*, and *MPL* mutational analyses and evaluations of mutational burden (variant allele frequency) were performed as previously described.^5^

### 2.3 RNA‐sequencing and data analysis

The evaluation of the expression of *PD‐L1/2* and other selected genes is based on a large RNA‐sequencing data set published in Schischlik et al.^27^ Further details are available in Appendix S1.

### 2.4 Targeted DNA‐sequencing and microarray analysis

Methods used for targeted DNA sequencing with the TruSight Myeloid Sequencing Panel (Illumina, San Diego, CA) and microarray analysis using Genome‐Wide Human SNP 6.0 arrays (Affymetrix, San Diego, CA, USA) are described in the Appendix S1.

### 2.5 Cell lines and in vitro studies

In vitro studies were performed using HEL, SET‐2, and UT‐7 cell lines. UT‐7 cells were engineered to express various *CALR* mutants using CRISPR/Cas9 technology as described recently.^28^ Methods related to in vitro studies as well as ^3^H‐thymidine incorporation assay are described in the Appendix S1.

### 2.6 Evaluation of expression of PD‐L1/2 and PD‐1 on primary MPN cells and cell lines

Heparinized BM or PB samples or cell lines were incubated with various combinations of monoclonal antibodies (Table S2) at room temperature in the dark for 15 min. As normal control, we used commercially available BM cells from healthy donors (Lonza, Basel, CH) as previously described.^29^ After incubation, erythrocytes were lysed using fluorescence‐activated cell sorting (FACS)‐Lysing Solution (BD Biosciences, San José, CA, USA). Cells were washed and analyzed by flow cytometry using FACSCanto II (BD Biosciences, San José, CA, USA) essentially as decribed.^30^ FlowJo software (version 8.8.7, TreeStar, Ashland, OR, USA) was used for data analysis. Antibody‐staining results were controlled by applying isotype‐matched control antibodies, and were expressed as either percentage of positive cells or as staining index, which represents the ratio of median fluorescence intensities obtained with the specific monoclonal antibody and the isotype‐matched control antibody. Phenotypic NSC were defined as CD34^+^CD45^dim^CD38^−^ while progenitors were defined as CD34^+^CD45^dim^CD38^+^ cells.^31^, ^32^ For detection of T cells, we used monoclonal antibodies against CD45, CD3, CD4, and CD8, while B cells were analyzed using CD19 antibody and NK cells by using a CD56 antibody. Gating strategies were applied as described previously.^30^

To assess the effects of drugs on PD‐L1 expression, primary MPN MNC from 6 *JAK2*‐V617F‐positive patients were incubated in medium or medium containing interferon‐gamma (IFN‐γ) (200 U/mL) in the absence or presence of the BET (Bromodomain and Extra‐Terminal proteins) inhibitor JQ1 (2500 nM), the BET degrader dBET6 (100 nM), ruxolitinib (2500 nM) or dimethyl sulfoxide (DMSO) (control) at 37°C for 24 h. JQ1 and ruxolitinib were obtained from Selleckchem (Houston, TX, USA) and dBET6 from Aobious (Gloucester, MA, USA). Then, cells were harvested and CD34^+^CD45^dim^CD38^−^ MPN stem cells were examined for expression of PD‐L1 by multicolor flow cytometry as described above. To assess drug effects on PD‐L1 expression in cell lines, cells were incubated in IFN‐γ (200 U/mL) in the absence or presence of DMSO, JQ1 (100–250 nM), dBET6 (50–500 nM), or ruxolitinib (50–250 nM) at 37°C for 24 h. Then, cells were examined for expression of PD‐L1 by flow cytometry.

### 2.7 Linkage analysis, association analysis, and haplotype‐based expression analysis

Linkage analysis for the JAK2 and PD‐L1 loci was performed using the LDpair and LDmatrix functions as implemented in the LDlink web‐based toolset (https://ldlink.nci.nih.gov/). Haplotype‐based association analyses were performed using previously published SNP‐array data from 272 MPN patients from Vienna, Austria^33^ in conjunction with 1620 Bavarian controls from the German Cooperative Health Research in the Region of Augsburg cohort (KORA).^34^ Cohort analyses of genetic data, including haplotype determination were performed using PLINK.^35^ Association analyses based on haplotype frequency distributions were performed using Fisher's exact test as implemented in R.^36^ Haplotype‐based comparative expression analysis was performed for MPN patients with both SNP‐array and RNA‐Seq data available. Statistical evaluation was performed using unpaired *t*‐tests as implemented in Prism (version 8.0.0, GraphPad Software, La Jolla, CA, USA).

### 2.8 Statistical analyses

Statistical analyses were performed using Prism software (Graphpad Software). For comparison of marker expression in various subgroups of patients, analysis of variance was applied. Significance levels in differences in expression of markers and targets on stem‐ and progenitor cells in various groups of patients were analyzed by Student's t‐test with or without Welch's correction. *p* < .05 was considered statistically significant.

## 3 RESULTS

### 3.1 *PD‐L1* is overexpressed in myeloid cells in MPN and is highest in PV

**FIGURE 1:**
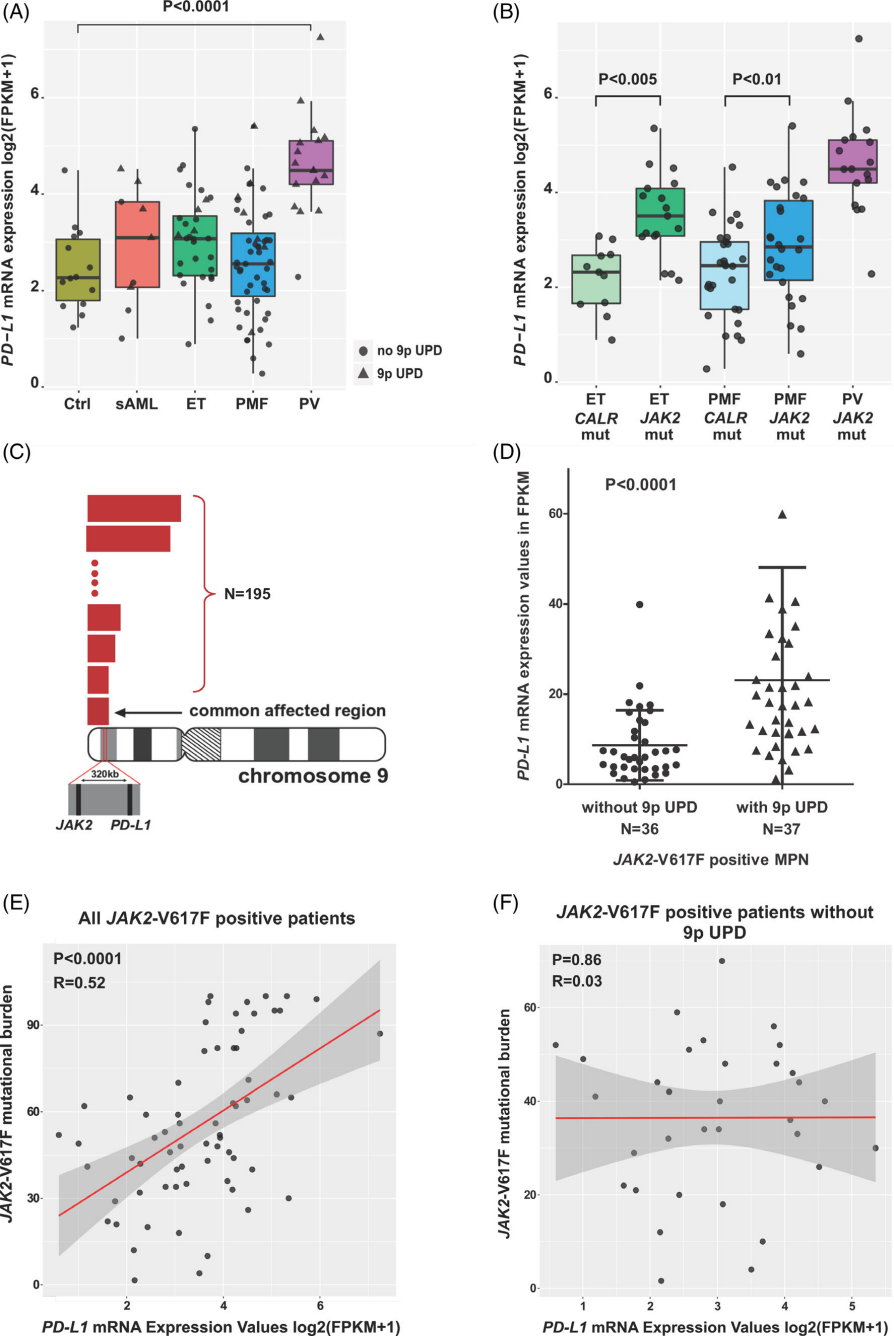
RNA‐sequencing reveals upregulation of *PD‐L1* in myeloid cells of patients with polycythemia vera and the role of chromosome 9p UPD in *PD‐L1* upregulation in MPN. (A) The box plots show the median (bold horizontal line), interquartile range (box), and total range (whiskers) of *PD‐L1* mRNA expression levels detected by RNA‐sequencing of granulocyte samples from MPN and secondary AML patients and healthy donors. Patients with PV displayed significant upregulation of *PD‐L1* expression compared to healthy donors and other MPN phenotypes; (B) RNA‐sequencing of granulocyte samples from MPN patients with ET, PMF, and PV revealed higher *PD‐L1* expression in *JAK2*‐V617F mutant patients than in *CALR* mutation‐driven ET and PMF. The box plots show the median (bold horizontal line), interquartile range (box), and total range (whiskers) of *PD‐L1* mRNA expression; (C) Chromosome 9p UPD, detected in 195 MPN patient samples using Human Whole‐genome Affymetrix 6.0 SNP arrays, always targets both *JAK2* and *PD‐L1* genes, despite a more centromeric position of *PD‐L1*. (D) *PD‐L1* mRNA expression in granulocytes, evaluated by RNA‐sequencing, is significantly higher in *JAK2*‐V617F‐positive patients who in addition carry a chromosome 9p UPD than in *JAK2*‐V617F positive patients without this aberration (*p* < .0001). The horizontal line represents the mean ± standard deviation. (E) *PD‐L1* mRNA expression measured by RNA‐sequencing is significantly correlated with the granulocyte *JAK2*‐V617F mutational burden (*R* = 0.52; *p* < .0001). (F) When excluding MPN cases with chromosome 9p UPD from this analysis, the correlation between PD‐L1 mRNA expression and *JAK2*‐V617F mutational burden in granulocytes of MPN patients is lost (*R* = 0.03; *p* = .9). Ctrl, control; ET, essential thrombocythemia; mut, mutant; PMF, primary myelofibrosis; PV, polycythemia vera; sAML, secondary acute myeloid leukemia; UPD, uniparental disomy [Color figure can be viewed at wileyonlinelibrary.com]

To determine the expression levels of *PD‐L1* and *PD‐L2* in MPN cells, we analyzed the whole‐transcriptome RNA‐sequencing data set of a well‐characterized cohort of 104 patients with chronic phase MPN, 9 patients with post‐MPN sAML, and 14 healthy controls (Figures 1A,B and S1,2).^27^ The MPN cohort included 55 patients with PMF, 32 with ET, and 17 with PV. The samples were selected based on the highest possible burden of the disease‐driving mutations in granulocytes in order to ensure that the vast majority of the cells examined would represent the MPN clone. In addition, we chose a balanced number of *JAK2*‐V617F and *CALR*‐mutated cases among ET and PMF patients. We observed a significantly higher expression level of *PD‐L1* mRNA in patients with PV compared to healthy controls (*p* < .0001; Figure 1A). To assess the effect of the disease‐driving mutations on *PD‐L1* expression in granulocytes, we divided the patient cohorts into subsets based on disease‐driving mutations and diagnosis (Figure 1B). Among both ET and PMF patients, *JAK2*‐V617F positive cases showed higher *PD‐L1* mRNA expression in granulocytes compared to *CALR*‐mutated MPN patients (*p* < .005 and *p* < .01, respectively; Figure 1B). Patients with PV who were all carrying the *JAK2*‐V617F mutation, displayed the highest expression levels of *PD‐L1* mRNA in neoplastic cells.

Targeted sequencing of a gene panel relevant for myeloid malignancies was performed for 77 patients of the cohort used for whole‐transcriptome sequencing. The most common mutations besides the disease drivers were mutations in *TET2* in 14.3%, *DNMT3A* in 28.6%, and *SF3B1* in 10.4% of the patients. However, presence of these additional mutations did not show an effect on *PD‐L1* expression in MPN cells (data not shown).

*PD‐L2*, encoding the other ligand of PD‐1, was expressed at very low levels in MPN cells in all samples analyzed, with PV patients displaying slightly increased expression levels compared to the other patient groups (Figure S2).

### 3.2 *PD‐L1* expression correlates with the *JAK2*‐V617F mutational burden and is higher in patients with UPD of chromosome 9p

As *PD‐L1* and *JAK2* are both located on the 9p24.1 locus (320 kb apart; Figure 1C), we hypothesized that the presence of chromosome 9p UPD has an effect on the observed *PD‐L1* expression in MPN cells, which would explain why MPN cells in patients with PV have higher *PD‐L1* expression levels compared to other disease entities. To test this hypothesis, we analyzed a previously published cohort of MPN patients for whom we had available data from Genome‐wide Human Affymetrix 6.0 SNP arrays.^33^ We detected chromosome 9p UPD in 195 MPN patients, while 15 patients carried three copies of chromosome 9p (9p gains), out of 408 patients analyzed. In all of the identified MPN cases with chromosome 9p aberrations, we observed that the commonly affected region invariably covers both, the *JAK2* and *PD‐L1* genes (Figure 1C). As *PD‐L1* has a more centromeric position, it is possible that this gene represents the second target of chromosome 9p UPD in MPN.

To determine the presence of 9p UPD in our cohort of patients characterized by RNA‐sequencing, we analyzed the SNP‐array data that was available for 73 *JAK2*‐V617F positive patients, and found that 37 patients carried the 9p UPD, while in 36 patients this aberration was absent. To assess the effect of the 9p UPD on *PD‐L1* expression in MPN cells, we compared the *PD‐L1* mRNA expression between *JAK2*‐V617F‐positive patients with and without 9p UPD, and observed a significantly higher expression of *PD‐L1* in MPN cells in patients with 9p UPD (*p* < .0001; Figure 1D). In addition, we found that the presence of the 9p UPD leads to higher *PD‐L1* expression in various MPN subsets (Figure S3). Next, we performed correlation studies, which revealed that *PD‐L1* levels correlate with the *JAK2*‐V617F mutational burden in granulocytes of MPN patients (*p* < .0001, *R* = 0.52; Figure 1E). The correlation of *PD‐L1* expression with the *JAK2*‐V617F mutational burden was lost when cases with 9p UPD were excluded from these analyses (*p* = .9, *R* = 0.03; Figure 1F).

### 3.3 Germline genetic variation at the *PD‐L1* gene locus affects MPN risk possibly through acting on *PD‐L1* expression

**FIGURE 2:**
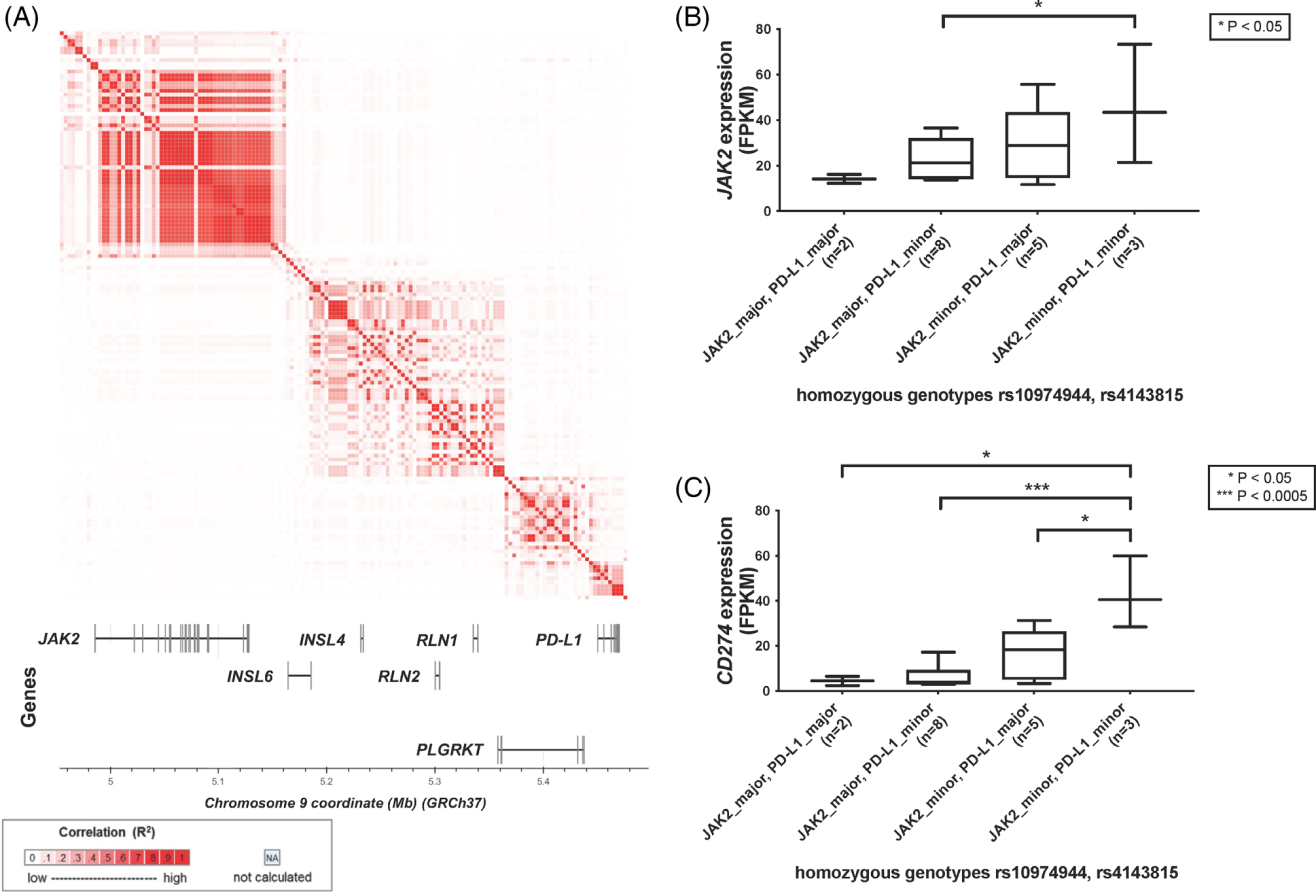
Evaluation of germline genetic variation at the PD‐L1 locus. (A) Linkage analyses for the *JAK2* and *PD‐L1* loci suggest the lack of strong LD between the loci (pairwise LD metrics for rs10974944‐rs4143815: *D*′ = 0.1171, R2 = 0.0112), while low correlations are observed all across the region. (B,C) Expression of *JAK2* (B) and *PD‐L1* (C) in MPN patients (*n* = 18) homozygous for both rs10974944 (*JAK2* locus) and rs4143815 (*PD‐L1* locus). Significance levels as determined by unpaired *t*‐tests are shown if *p* < .05 [Color figure can be viewed at wileyonlinelibrary.com]

Previous data have shown that a common haplotype referred to as GGCC (also: 46/1) haplotype preferentially acquires *JAK2*‐V617F and thus confers susceptibility for MPN.^37^ Our data suggest that the presence of chromosome 9p UPD might be relevant for *PD‐L1* upregulation also at the more distal *PD‐L1* locus. Therefore, we evaluated a possible role of germline genetic variation at the *PD‐L1* locus and its interplay with the GGCC haplotype at the distal *JAK2* locus. We performed linkage analysis, including the GGCC haplotype and an SNP within the 3’UTR of *PD‐L1* (rs4143815), as there were no suitable coding SNPs available. We did not observe a strong linkage disequilibrium (LD) between the *JAK2* and *PD‐L1* loci (D′ = 0.1171, *R* ^2^ = .0112), however, the presence of a haplotype block (genomic region with low recombination rate and low haplotype diversity) spanning both loci at weak LD could not be excluded (Figure 2A). Next, we determined the frequencies of the possible JAK2/PD‐L1 haplotypes in a large SNP‐array‐typed MPN cohort (*n* = 272)^27^ and a population‐matched non‐MPN control cohort (*n* = 1620; Table S3). Haplotype‐based association analyses on the observed counts suggested the *PD‐L1* rs4143815 minor allele as risk factor for MPN susceptibility independent of the *JAK2* GGCC haplotype tagged by rs10974944 (Table 1). This was observed on both GGCC risk (rs10974944 minor allele; *p* = .04) and GGCC protective (rs10974944 major allele; *p* = .02) backgrounds (Table 1). These observations are supported by a GGCC‐independent effect of the PD‐L1 haplotype on expression at the larger locus, including both *JAK2* and *PD‐L1*, although a modest number of informative samples was available for this analysis (Figure 2B,C). Specifically, the presence of the *PD‐L1* rs4143815 minor allele results in increased JAK2 expression on both JAK2 GGCC protective (major) and risk (minor) background, albeit lacking formal statistical significance (Figure 2B). A similar overall effect can be observed for PD‐L1 expression, where the presence of the *PD‐L1* rs4143815 minor allele on GGCC risk background results in increased *PD‐L1* expression (*p* = .03; Figure 2C).

**TABLE 1:** Association analysis for an MPN patient cohort (*n* = 272) versus a non‐MPN control cohort (*n* = 1620) for haplotypes, including both *JAK2* (rs10974944) and *PD‐L1* (rs4143815); results from homozygous calls at both loci are shown

Reference haplotype	Test haplotype	OR	95% CI lower	95% CI upper	*p*‐value	Effect tested
*JAK2*_major, *PD‐L1*_major	*JAK2*_major, *PD‐L1*_minor	2.15	0.94	4.61	.04	*PD‐L1* minor allele alone
*JAK2*_major, *PD‐L1*_major	*JAK2*_minor, *PD‐L1*_major	7.24	3.85	13.62	2.74E−10	*JAK2* minor allele alone
*JAK2*_major, *PD‐L1*_major	*JAK2*_minor, *PD‐L1*_minor	18.83	8.4	43.66	4.23E−14	*JAK2* and *PD‐L1* minor alleles combined
*JAK2_*minor, *PD‐L1*_minor	*JAK2*_minor, *PD‐L1*_major	0.39	0.15	0.94	.02	*PD‐L1* major allele alone
*JAK2*_minor, *PD‐L1*_minor	*JAK2*_major, *PD‐L1*_minor	0.11	0.04	0.31	1.78E−06	*JAK2* major allele alone
*JAK2*_minor, *PD‐L1*_minor	*JAK2*_major, *PD‐L1*_major	0.05	0.02	0.12	4.23E−14	*JAK2* and *PD‐L1* major alleles combined

### 3.4 PD‐L1 is expressed on the cell surface of NSC in MPN patients

**FIGURE 3:**
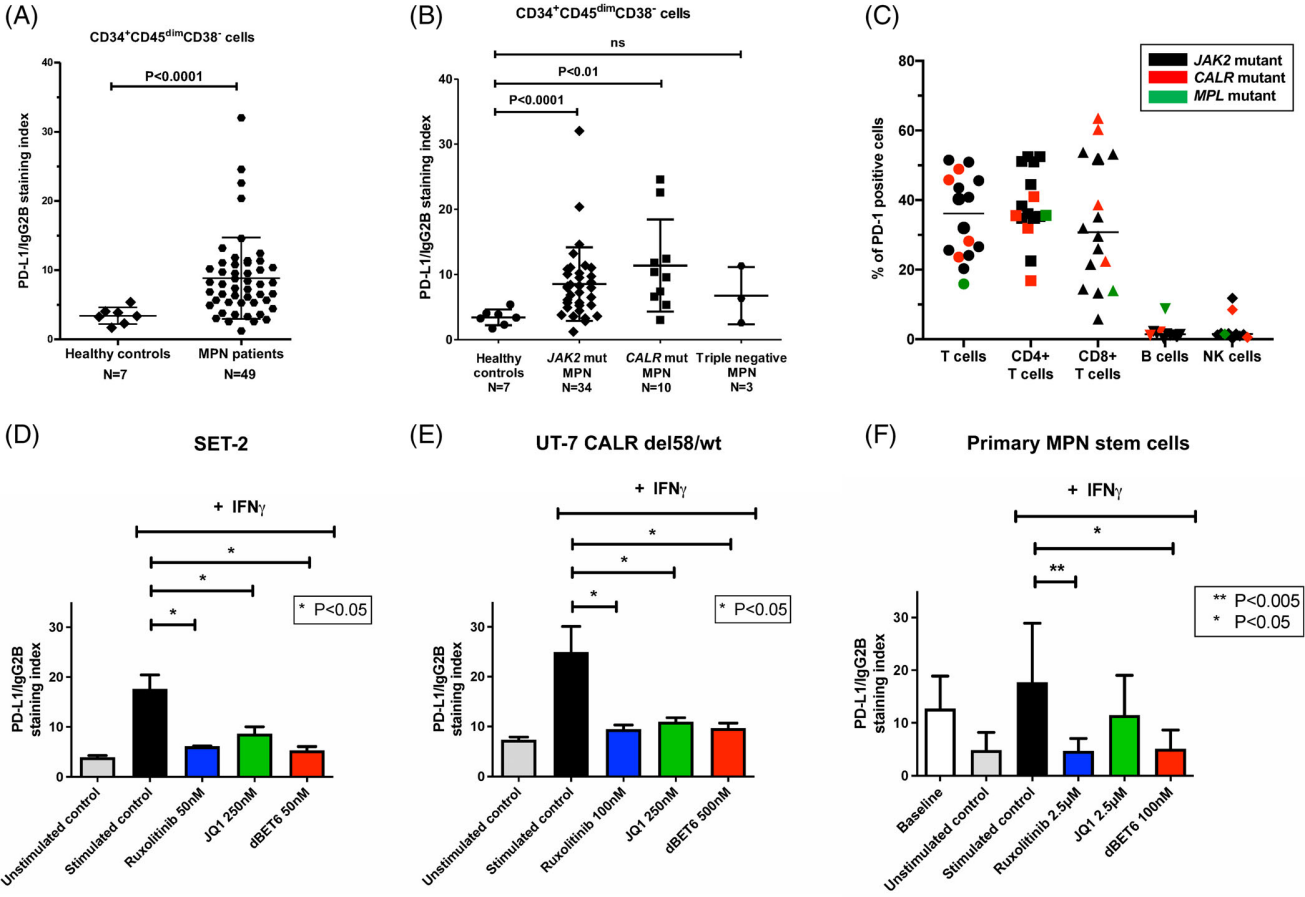
PD‐L1 is upregulated on the surface of stem cells isolated from bone marrow samples of MPN patients and can be downregulated by ruxolitinib and dBET6. (A,B) PD‐L1 expression was assessed by multicolor flow cytometry on CD34^+^CD45^dim^CD38^−^ putative neoplastic stem cells isolated from the bone marrow of MPN patients (*N* = 49) or healthy donors (*N* = 7). The horizontal line represents the mean ± standard deviation. (B) Both *JAK2* and *CALR* mutant MPN patients showed an upregulation of PD‐L1 on stem cells when compared to healthy donors (*p* < .001 and *p* < .01, respectively). The horizontal line represents the mean ± standard deviation. (C) Expression of PD‐1 on T cells, B cells, and NK cells from fresh bone marrow samples of MPN patients was assessed using multicolor flow cytometry. The results are shown as percentage of PD‐1 positive cells and each datapoint represents one patient, while the horizontal line represents the median value. PD‐1 was expressed on both CD4^+^ and CD8^+^ T cells, but was not found to be expressed on B and NK cells of the majority of the samples analyzed. (D) SET‐2 and UT‐7 CALR del58/wt (E) cells were incubated with medium or medium containing 200 U/mL of IFN‐γ with or without indicated concentrations of ruxolitinib, JQ1, or dBET6. Drug concentrations were selected at IC_20_‐IC_30_ for each cell line. Expression of PD‐L1 was evaluated using flow cytometry upon 24 h of incubation. The expression of PD‐L1 is shown as the staining index, which represents the ratio of median fluorescence intensity of PD‐L1 and matched isotype control. The experiments were performed in triplicate and graphs represent mean ± standard deviation. (F) Primary MNC from six independent *JAK2*‐V617F positive MPN patients were incubated with medium or medium containing 200 U/mL of IFN‐γ with or without the indicated concentrations of ruxolitinib, JQ1, or dBET6 for 24 h. Upon incubation, PD‐L1 expression on CD34^+^CD45^dim^CD38^−^ cells was evaluated using multicolor flow cytometry. The expression of PD‐L1 is shown as the staining index, which represents the ratio of median fluorescence intensity of PD‐L1 antibody and the isotype‐matched control antibody. The graph represents mean ± standard deviation of six independent experiments [Color figure can be viewed at wileyonlinelibrary.com]

As we demonstrated the PD‐L1 mRNA upregulation in myeloid cells of MPN patients, we wanted to confirm these findings at the protein level. To investigate if the phenotypically defined (CD34^+^CD45^dim^CD38^−^) NSC in MPN patients express PD‐L1 on their cell surface, we analyzed cells isolated from fresh BM samples of 49 MPN patients and 7 healthy controls by flow cytometry (Figures 3A,B and S4). PD‐L1 surface expression was assessed in both, CD34^+^CD45^dim^CD38^−^ and CD34^+^CD45^dim^CD38^+^ MPN cell populations. Stem cells and progenitor cells in MPN patients showed a significantly higher surface expression of PD‐L1 compared to stem and progenitor cells in healthy controls (*p* < .0001) (Figures 3A and S5A). Both *JAK2*‐mutated and *CALR*‐mutated MPN patients showed significantly higher surface expression of PD‐L1 on NSC and progenitor cells compared to normal stem and progenitor cells (Figures 3B and S5B). By contrast, PD‐L1 expression on CD34^+^cell subsets in the three triple‐negative MPN patients tested, did not significantly differ from PD‐L1 levels detected on CD34^+^ cells in healthy controls (Figures 3B and S5B). Among the MPN patients analyzed by flow cytometry, 7 were diagnosed with PV, 28 with ET, 6 with PMF, and 5 with post‐MPN AML. Surprisingly, no significant difference in the surface expression of PD‐L1 on NSC measured by flow cytometry was observed among the four different phenotypes (Figures S4 and S6). This may be explained by low *JAK2*‐V617F burden in the seven PV patients we analyzed by FACS. NSC of MPN patients did not express PD‐L2 on the cell surface as measured by flow cytometry (Figure S7A,C) and the same was observed for progenitor cells (Figure S7B).

Finally, we found that both CD4^+^ and CD8^+^ T cells isolated from fresh BM samples of MPN patients (N = 16) express the PD‐L1 receptor PD‐1, and that this expression is not influenced by the presence of the MPN disease‐driving mutations *JAK2*, *CALR*, or *MPL* (Figures 3C and S8). In almost all MPN patients tested (N = 12), B cells and NK cells did not express PD‐1 on their cell surface (Figures 3C and S8).

### 3.5 PD‐L1 expression in MPN (stem) cells can be modulated by ruxolitinib and BRD4‐targeting drugs and both agents suppress proliferation of MPN cells

We evaluated the effects of ruxolitinib and of the BRD4‐targeting drugs JQ1 and dBET6 on IFN‐γ‐induced expression of PD‐L1 in the *JAK2‐*V617F‐positive cell lines HEL and SET‐2, and UT‐7 cells carrying *CALR* mutations. In all tested *JAK2*‐V617F‐ and *CALR*‐mutated cell lines, ruxolitinib, JQ1, and dBET6 were found to downregulate IFN‐γ‐induced PD‐L1 expression after 24 h as measured by flow cytometry, while no significant difference was observed in UT‐7 wild type cells upon treatment (Figures 3D,E and S9). We also examined drug effects on primary MPN stem cells obtained from six patients with *JAK2*‐V617F‐positive MPN. We found that ruxolitinib and dBET6 completely suppress IFN‐γ‐induced expression of PD‐L1 in phenotypically defined (CD34^+^CD45^dim^CD38^−^) MPN NSC, while JQ1 did not show a significant effect (Figure 3F). Interestingly, ex vivo culturing of primary MPN stem cells in Roswell Park Memorial Institute (RPMI) medium for 24 h (in the absence of IFN‐γ) led to reduced PD‐L1 levels compared to baseline levels measured in stem cells in fresh BM samples. Finally, we were able to show that JQ1 and dBET6 dose‐dependently suppress the growth of primary MPN cells (Figure S10) and the same effect was observed with JQ1 and dBET6 in the MPN‐related cell lines HEL, SET‐2, and UT‐7 cells carrying *CALR* mutations (Figure S11).

## 4 DISCUSSION

Previously published data have demonstrated that *JAK2*‐V617F‐positive MPN cells display PD‐L1, a major immune checkpoint antigen mediating immunological resistance in neoplastic cells in diverse hematologic malignancies.^14^ So far, little is known about the regulation and function of expression of PD‐L1 in MPN cells. We here show that PD‐L1 expression in MPN cells is highest in patients with PV, correlates with the *JAK2*‐V617F burden, and is associated with chromosome 9p UPD. Moreover, we show that PD‐L1 is not only expressed on mature myeloid cells in MPN, but also on the disease‐initiating NSC in all MPN patients tested. Finally, our data show that JAK2‐targeting and BRD4/MYC‐targeting drugs counteract the IFN‐γ‐induced expression of PD‐L1 on MPN cells and MPN NSC.

Although responses to PD‐L1/PD‐1‐targeting therapy have been documented even in cancers where PD‐L1 expression was low, high PD‐L1 expression is often used as a biomarker and predictor of response to PD‐L1/PD‐1‐targeting therapy in several types of cancer, including Hodgkin lymphoma.^21^, ^23^, ^38^, ^39^, ^40^ Patients with Hodgkin lymphoma often carry amplifications of the chromosome 9p24.1 locus containing *PD‐L1*, *PD‐L2*, and *JAK2* genes, leading to high PD‐L1 and PD‐L2 protein expression.^41^, ^42^, ^43^ In MPN, chromosome 9p is affected by UPD most commonly in PV, but also in patients with PMF and ET.^26^ The 9p UPD is considered to be a late event in the clonal evolution of MPN, occurring after the acquisition of *JAK2*‐V617F and leading to amplification of oncogenic mutation to homozygosity and an increase in mutational burden.^25^ However, several studies have provided evidence that 9p UPD can occur prior to *JAK2*‐V617F in up to 10% of MPN patients, indicating that there might be a second target of this chromosomal aberration.^44^, ^45^ We here show that *PD‐L1* is always affected by chromosome 9p aberrations in MPN. The fact that *PD‐L1* expression in MPN cells correlates with the *JAK2*‐V617F mutational burden and that among *JAK2*‐V617F positive patients, those with homozygous *JAK2*‐V617F mutation display higher *PD‐L1* expression, indicate that the mutant JAK2 dosage and subsequent activation of the JAK–STAT pathway, lead to PD‐L1 upregulation, which is in line with the results published by Prestipino et al.^14^

Acquisition of chromosome 9p UPD and subsequent upregulation of PD‐L1 expression on affected cells may contribute to a positive selection of *JAK2*‐V617F homozygous stem cells. As we are not capable of modeling chromosome 9p UPD without the presence of *JAK2*‐V617F, we attempted to evaluate the influence of germline genetic factors on expression of *PD‐L1*. While we did not observe a strong LD between the GGCC haplotype and the 3’UTR of *PD‐L1*, we could show that carriers of the minor allele of the *PD‐L1* variant rs4143815 are enriched in an MPN cohort. Of note, this enrichment was observed independent of the GGCC haplotype, establishing a novel risk factor for MPN susceptibility, albeit at a modest effect size as compared to the adjacent *JAK2* GGCC risk locus. While the GGCC haplotype reflects strongly increased *PD‐L1* expression levels, either directly through germline regulatory mechanisms or through increased acquisition of *JAK2*‐V617F, our results demonstrate an additive, but independent effect of the “PD‐L1 risk haplotype” tagged by the minor allele of rs4143815 on *PD‐L1* expression. As our analyses were performed in an MPN cohort, it remains to be determined whether similar effects can be observed also in cancer cells without *JAK2*‐V617F mutation. Overall, these data provide first evidence for a role of germline factors at the *PD‐L1* locus in MPN pathogenesis independent of the *JAK2* GGCC risk haplotype. However, potential presence of a haplotype block at weak LD prevents a full dissection of the two loci by genetic means, thus requiring functional studies.

We demonstrated that stem and progenitor cells from MPN patients express high levels of PD‐L1, regardless of the *JAK2* and *CALR* mutational status. This finding might stem from the external factors present in the BM microenvironment, which can affect PD‐L1 expression. The involvement of the IFN‐γ signaling through JAK1/2‐STAT axis in expression of PD‐L1 has been well documented.^46^, ^47^ Altered expression of cytokines is considered to be a hallmark of MPN and measurable levels of IFN‐γ in the plasma or sera of patients with MPN have been reported in several studies, albeit with conflicting results.^48^, ^49^, ^50^ We could demonstrate that the PD‐L1 expression in CD34^+^CD45^dim^CD38^−^ stem cells of *JAK2*‐V617F positive MPN patients spontaneously decreases when the cells are incubated ex vivo in RPMI medium for 24 h, and that the stimulation with IFN‐γ restores PD‐L1 expression to normal baseline levels. Therefore, we cannot rule out that the difference in PD‐L1 expression among the MPN phenotypes, or between the stem and progenitor cell populations and granulocytes in the case of *CALR* positive patients could at least in part be explained by extrinsic factors, such as different IFN‐γ levels. An alternative explanation would be that PD‐L1 levels are differentially regulated by cytokines or oncogenic machineries in different types of cells. PD‐L1 upregulation on progenitor cells and stem cells of *CALR* positive MPN patients is in line with the findings of Bozkus et al. who demonstrated that these patients develop T cells specific for the mutant CALR and that targeting the PD‐L1/PD‐1 interaction can lead to restoration of this T‐cell response against mutant CALR in MPN patients.^51^

The ability of BET inhibitors, such as JQ1, to suppress constitutive or IFN‐γ induced PD‐L1 expression in certain cancer cell lines as well as stem cells of chronic myeloid leukemia patients were previously described.^52^, ^53^ In line with these results, we show that treatment with the JAK2 inhibitor ruxolitinib or the BET‐degrader dBET6, but not BET inhibitor JQ1, leads to downregulation of PD‐L1 expression in CD34^+^CD45^dim^CD38^−^ stem cells in *JAK2‐*V617F positive MPN patients, while all three drugs exerted inhibitory effects in *JAK2* and *CALR*‐mutated cell lines. It remains unknown whether these drug effects on PD‐L1 expression play a role in vivo in patients with MPN and may support or facilitate the cytoreductive capabilities of these agents against MPN (stem) cells. In this regard, it is worth noting that ruxolitinib exerts modest effects on depleting MPN cells and reducing the *JAK2*‐V617F mutant allele burden in MPN patients (only 10%–20% reduction) and also showed little effects on disease progression to post‐MPN sAML.^54^, ^55^ It is also of interest to note that BET protein bromodomain targeting agents were previously shown to be highly active against post‐MPN sAML cells, and to exert a synergistic effect with ruxolitinib.^56^, ^57^ As previous reports also demonstrated synergistic effects of anti‐PD‐1 antibodies with BET inhibitor JQ1,^52^ our data indicates that such applications should be assessed in future studies in the context of MPN.

In conclusion, our data show that PD‐L1 is expressed abundantly in MPN cells, including MPN‐initiating phenotypically defined CD34^+^CD45^dim^CD38^−^ NSC. In addition, we demonstrate that PD‐L1 levels are highest in neoplastic cells in patients with PV, correlate with the *JAK2*‐V617F burden and with chromosome 9p UPD, and are promoted by IFN‐γ exposure through a BRD4/MYC‐dependent pathway. We also provide first evidence that germline genetic factors at the *PD‐L1* locus contribute to MPN susceptibility independently of the GGCC (46/1) risk haplotype. Since PD‐L1 is a major resistance‐mediating immune checkpoint, these data may have clinical implications and may pave the way for the development and application of new PD‐L1‐blocking therapies in MPN.

## CONFLICTS OF INTEREST

R.K. has received honoraria from and served on the advisory board of AOP Orphan Pharmaceuticals AG, has received honoraria from Pharma Essentia, and has equity ownership in MyeloPro Diagnostics and Research GmbH. H.G. has been a consultant to and received honoraria and research funding from AOP Orphan Pharmaceuticals AG; has received honoraria from Novartis, Celgene, and Janssen‐Cilag; has been a consultant to Roche, MyeloPro Diagnostics, and Research GmbH; and has received personal fees from PharmaEssentia. P.V. received honoraria from Novartis, Incyte and BMS/Celgene. The remaining authors declare no competing financial interests.

## Supporting information


**Appendix** **S1** Supporting Information

## Data Availability

The RNA‐sequencing and microarray data have been deposited in the following repositories: European Genome‐phenome Archive; Accession ID: EGAS00001003486 and Array express; Accession ID: E‐MTAB‐1845.

## References

[1] Barosi G . An immune dysregulation in MPN. Curr Hematol Malig Rep. 2014;9:331‐339. 25139710 10.1007/s11899-014-0227-0

[2] Vainchenker W , Kralovics R . Genetic basis and molecular pathophysiology of classical myeloproliferative neoplasms. Blood. 2017;129:667‐679. 28028029 10.1182/blood-2016-10-695940

[3] Baxter EJ , Scott LM , Campbell PJ , et al. Acquired mutation of the tyrosine kinase JAK2 in human myeloproliferative disorders. Lancet. 2005;365:1054‐1061. doi:10.1016/S0140-6736(05)71142-9 15781101

[4] James C , Ugo V , Le Couedic JP , et al. A unique clonal JAK2 mutation leading to constitutive signalling causes polycythaemia vera. Nature. 2005;434:1144‐1148. doi:10.1038/nature03546 15793561

[5] Kralovics R , Passamonti F , Buser AS , et al. A gain‐of‐function mutation of JAK2 in myeloproliferative disorders. N Engl J Med. 2005;352:1779‐1790. doi:10.1056/NEJMoa051113 15858187

[6] Levine RL , Wadleigh M , Cools J , et al. Activating mutation in the tyrosine kinase JAK2 in polycythemia vera, essential thrombocythemia, and myeloid metaplasia with myelofibrosis. Cancer Cell. 2005;7:387‐397. doi:10.1016/j.ccr.2005.03.023 15837627

[7] Klampfl T , Gisslinger H , Harutyunyan AS , et al. Somatic mutations of calreticulin in myeloproliferative neoplasms. N Engl J Med. 2013;369:2379‐2390. doi:10.1056/NEJMoa1311347 24325356

[8] Pardanani AD , Levine RL , Lasho T , et al. MPL515 mutations in myeloproliferative and other myeloid disorders: a study of 1182 patients. Blood. 2006;108:3472‐3476. doi:10.1182/blood-2006-04-018879 16868251

[9] Pikman Y , Lee BH , Mercher T , et al. MPLW515L is a novel somatic activating mutation in myelofibrosis with myeloid metaplasia. PLoS Med. 2006;3:e270. doi:10.1371/journal.pmed.0030270 16834459 PMC1502153

[10] Abdulkarim K , Girodon F , Johansson P , et al. AML transformation in 56 patients with Ph‐ MPD in two well defined populations. Eur J Haematol. 2009;82:106‐111. doi:10.1111/j.1600-0609.2008.01163.x 19134023

[11] Asher S , McLornan DP , Harrison CN . Current and future therapies for myelofibrosis. Blood Rev. 2020;42:100715. doi:10.1016/j.blre.2020.100715 32536371

[12] Guglielmelli P , Vannucchi AM . Current management strategies for polycythemia vera and essential thrombocythemia. Blood Rev. 2020;42:100714. doi:10.1016/j.blre.2020.100714 32546373

[13] Braun LM , Zeiser R . Immunotherapy in myeloproliferative diseases. Cell. 2020;9(6):1559. doi:10.3390/cells9061559 PMC7349594 32604862

[14] Prestipino A , Emhardt AJ , Aumann K , et al. Oncogenic JAK2(V617F) causes PD‐L1 expression, mediating immune escape in myeloproliferative neoplasms. Sci Transl Med. 2018;10:eaam7729. doi:10.1126/scitranslmed.aam7729 PMC6034655 29467301

[15] Wang JC , Chen C , Kundra A , et al. Programmed cell death receptor (PD‐1) ligand (PD‐L1) expression in Philadelphia chromosome‐negative myeloproliferative neoplasms. Leuk Res. 2019;79:52‐59. doi:10.1016/j.leukres.2019.02.010 30851544

[16] Freeman GJ , Long AJ , Iwai Y , et al. Engagement of the PD‐1 immunoinhibitory receptor by a novel B7 family member leads to negative regulation of lymphocyte activation. J Exp Med. 2000;192:1027‐1034. doi:10.1084/jem.192.7.1027 11015443 PMC2193311

[17] Latchman Y , Wood CR , Chernova T , et al. PD‐L2 is a second ligand for PD‐1 and inhibits T cell activation. Nat Immunol. 2001;2:261‐268. doi:10.1038/85330 11224527

[18] Curiel TJ , Wei S , Dong H , et al. Blockade of B7‐H1 improves myeloid dendritic cell‐mediated antitumor immunity. Nat Med. 2003;9:562‐567. doi:10.1038/nm863 12704383

[19] Zou W , Wolchok JD , Chen L . PD‐L1 (B7‐H1) and PD‐1 pathway blockade for cancer therapy: mechanisms, response biomarkers, and combinations. Sci Transl Med. 2016;8(328):328rv4. doi:10.1126/scitranslmed.aad7118 PMC4859220 26936508

[20] Cha JH , Chan LC , Li CW , Hsu JL , Hung MC . Mechanisms controlling PD‐L1 expression in cancer. Mol Cell. 2019;76(3):359‐370. doi:10.1016/j.molcel.2019.09.030 31668929 PMC6981282

[21] Garon EB , Rizvi NA , Hui R , et al. Pembrolizumab for the treatment of non‐small‐cell lung cancer. N Engl J Med. 2015;372:2018‐2028. doi:10.1056/NEJMoa1501824 25891174

[22] Wolchok JD , Kluger H , Callahan MK , et al. Nivolumab plus ipilimumab in advanced melanoma. N Engl J Med. 2013;369:122‐133. doi:10.1056/NEJMoa1302369 23724867 PMC5698004

[23] Topalian SL , Hodi FS , Brahmer JR , et al. Safety, activity, and immune correlates of anti‐PD‐1 antibody in cancer. N Engl J Med. 2012;366:2443‐2454. doi:10.1056/NEJMoa1200690 22658127 PMC3544539

[24] Brahmer JR , Tykodi SS , Chow LQ , et al. Safety and activity of anti‐PD‐L1 antibody in patients with advanced cancer. N Engl J Med. 2012;366:2455‐2465. doi:10.1056/NEJMoa1200694 22658128 PMC3563263

[25] Kralovics R , Guan Y , Prchal JT . Acquired uniparental disomy of chromosome 9p is a frequent stem cell defect in polycythemia vera. Exp Hematol. 2002;30:229‐236. doi:10.1016/s0301-472x(01)00789-5 11882360

[26] Wang L , Wheeler DA , Prchal JT . Acquired uniparental disomy of chromosome 9p in hematologic malignancies. Exp Hematol. 2016;44:644‐652. doi:10.1016/j.exphem.2015.11.005 26646991 PMC5268131

[27] Schischlik F , Jager R , Rosebrock F , et al. Mutational landscape of the transcriptome offers putative targets for immunotherapy of myeloproliferative neoplasms. Blood. 2019;134:199‐210. doi:10.1182/blood.2019000519 31064751 PMC6624966

[28] Jia R , Balligand T , Atamanyuk V , et al. Hematoxylin binds to mutant calreticulin and disrupts its abnormal interaction with thrombopoietin receptor. Blood. 2020;137:1920‐1931. doi:10.1182/blood.2020006264 PMC8118640 33202418

[29] Herrmann H , Sadovnik I , Eisenwort G , et al. Delineation of target expression profiles in CD34+/CD38− and CD34+/CD38+ stem and progenitor cells in AML and CML. Blood Adv. 2020;4:5118‐5132. doi:10.1182/bloodadvances.2020001742 33085758 PMC7594398

[30] Eisenwort G , Sadovnik I , Schwaab J , et al. Identification of a leukemia‐initiating stem cell in human mast cell leukemia. Leukemia. 2019;33:2673‐2684. doi:10.1038/s41375-019-0460-6 30953030 PMC6839966

[31] Ivanov D , Milosevic Feenstra JD , Eisenwort G , et al. Phenotyping of disease‐initiating CD34^+^/CD38^─^ stem cells in BCR‐ABL1^─^ MPN reveals expression of multiple cytokine receptors and resistance‐related antigens. Blood. 2020;136(Supplement 1):53. doi:10.1182/blood-2020-140477

[32] Lysenko V , Wildner‐Verhey van Wijk N , Zimmermann K , et al. Enhanced engraftment of human myelofibrosis stem and progenitor cells in MISTRG mice. Blood Adv. 2020;4(11):2477‐2488. doi:10.1182/bloodadvances.2019001364 32502268 PMC7284099

[33] Klampfl T , Harutyunyan A , Berg T , et al. Genome integrity of myeloproliferative neoplasms in chronic phase and during disease progression. Blood. 2011;118:167‐176. doi:10.1182/blood-2011-01-331678 21531982

[34] Holle R , Happich M , Lowel H , Wichmann HE , Group MKS . KORA‐‐a research platform for population based health research. Gesundheitswesen. 2005;67(Suppl 1):S19‐S25. doi:10.1055/s-2005-858235 16032513

[35] Purcell S , Neale B , Todd‐Brown K , et al. PLINK: a tool set for whole‐genome association and population‐based linkage analyses. Am J Hum Genet. 2007;81:559‐575. doi:10.1086/519795 17701901 PMC1950838

[36] R Core Team . R: A Language and Environment for Statistical Computing. Foundation for Statistical Computing; 2013.

[37] Olcaydu D , Harutyunyan A , Jager R , et al. A common JAK2 haplotype confers susceptibility to myeloproliferative neoplasms. Nat Genet. 2009;41:450‐454. doi:10.1038/ng.341 19287385

[38] Herbst RS , Soria JC , Kowanetz M , et al. Predictive correlates of response to the anti‐PD‐L1 antibody MPDL3280A in cancer patients. Nature. 2014;515:563‐567. doi:10.1038/nature14011 25428504 PMC4836193

[39] Maleki Vareki S , Garrigos C , Duran I . Biomarkers of response to PD‐1/PD‐L1 inhibition. Crit Rev Oncol Hematol. 2017;116:116‐124. doi:10.1016/j.critrevonc.2017.06.001 28693793

[40] Ansell SM , Lesokhin AM , Borrello I , et al. PD‐1 blockade with nivolumab in relapsed or refractory Hodgkin's lymphoma. N Engl J Med. 2015;372:311‐319. doi:10.1056/NEJMoa1411087 25482239 PMC4348009

[41] Roemer MG , Advani RH , Ligon AH , et al. PD‐L1 and PD‐L2 genetic alterations define classical Hodgkin lymphoma and predict outcome. J Clin Oncol. 2016;34:2690‐2697. doi:10.1200/JCO.2016.66.4482 27069084 PMC5019753

[42] Roemer MGM , Redd RA , Cader FZ , et al. Major histocompatibility complex class II and programmed death ligand 1 expression predict outcome after programmed death 1 blockade in classic hodgkin lymphoma. J Clin Oncol. 2018;36:942‐950. doi:10.1200/JCO.2017.77.3994 29394125 PMC5877802

[43] Green MR , Monti S , Rodig SJ , et al. Integrative analysis reveals selective 9p24.1 amplification, increased PD‐1 ligand expression, and further induction via JAK2 in nodular sclerosing Hodgkin lymphoma and primary mediastinal large B‐cell lymphoma. Blood. 2010;116:3268‐3277. doi:10.1182/blood-2010-05-282780 20628145 PMC2995356

[44] Vilaine M , Olcaydu D , Harutyunyan A , et al. Homologous recombination of wild‐type JAK2, a novel early step in the development of myeloproliferative neoplasm. Blood. 2011;118:6468‐6470. doi:10.1182/blood-2011-08-372813 22161852

[45] Wang L , Swierczek SI , Lanikova L , et al. The relationship of JAK2(V617F) and acquired UPD at chromosome 9p in polycythemia vera. Leukemia. 2014;28:938‐941. doi:10.1038/leu.2014.20 24463469 PMC4532371

[46] Dong H , Strome SE , Salomao DR , et al. Tumor‐associated B7‐H1 promotes T‐cell apoptosis: a potential mechanism of immune evasion. Nat Med. 2002;8:793‐800. doi:10.1038/nm730 12091876

[47] Garcia‐Diaz A , Shin DS , Moreno BH , et al. Interferon receptor signaling pathways regulating PD‐L1 and PD‐L2 expression. Cell Rep. 2017;19:1189‐1201. doi:10.1016/j.celrep.2017.04.031 28494868 PMC6420824

[48] Vaidya R , Gangat N , Jimma T , et al. Plasma cytokines in polycythemia vera: phenotypic correlates, prognostic relevance, and comparison with myelofibrosis. Am J Hematol. 2012;87:1003‐1005. doi:10.1002/ajh.23295 22965887

[49] Obro NF , Grinfeld J , Belmonte M , et al. Longitudinal cytokine profiling identifies GRO‐alpha and EGF as potential biomarkers of disease progression in essential Thrombocythemia. Hemasphere. 2020;4:e371. doi:10.1097/HS9.0000000000000371 32647796 PMC7306314

[50] Tabarroki A , Rogers HJ , Visconte V , et al. The molecular and cytokine profile of triple‐negative (JAK2 V617F, JAK2 exon 12, MPL negative) myelofibrosis, a myeloproliferative neoplasm with distinct Clinico‐pathologic characteristics. Blood. 2012;120:3805. doi:10.1182/blood.V120.21.3805.3805

[51] Bozkus CC , Roudko V , Finnigan JP , et al. Shared Neoantigen‐induced T‐cell immunity directed against mutated Calreticulin in myeloproliferative neoplasms. Cancer Discov. 2019;9:1192‐1207. doi:10.1158/2159-8290.CD-18-1356 31266769 PMC6726533

[52] Hogg SJ , Vervoort SJ , Deswal S , et al. BET‐bromodomain inhibitors engage the host immune system and regulate expression of the immune checkpoint ligand PD‐L1. Cell Rep. 2017;18:2162‐2174. doi:10.1016/j.celrep.2017.02.011 28249162 PMC5340981

[53] Zhu H , Bengsch F , Svoronos N , et al. BET bromodomain inhibition promotes anti‐tumor immunity by suppressing PD‐L1 expression. Cell Rep. 2016;16:2829‐2837. doi:10.1016/j.celrep.2016.08.032 27626654 PMC5177024

[54] Vannucchi AM , Kiladjian JJ , Griesshammer M , et al. Ruxolitinib versus standard therapy for the treatment of polycythemia vera. N Engl J Med. 2015;372(5):426‐435. doi:10.1056/NEJMoa1409002 25629741 PMC4358820

[55] Verstovsek S , Mesa RA , Gotlib J , et al. A double‐blind placebo‐controlled trial of ruxolitinib for myelofibrosis. N Engl J Med. 2012;366(9):799‐807. doi:10.1056/NEJMoa1110557 22375971 PMC4822164

[56] Saenz DT , Fiskus W , Manshouri T , et al. BET protein bromodomain inhibitor‐based combinations are highly active against post‐myeloproliferative neoplasm secondary AML cells. Leukemia. 2017;31:678‐687. doi:10.1038/leu.2016.260 27677740 PMC5345582

[57] Saenz DT , Fiskus W , Qian Y , et al. Novel BET protein proteolysis‐targeting chimera exerts superior lethal activity than bromodomain inhibitor (BETi) against post‐myeloproliferative neoplasm secondary (s) AML cells. Leukemia. 2017;31:1951‐1961. doi:10.1038/leu.2016.393 28042144 PMC5537055

