# Supplementary material for: PD‐L1 overexpression correlates with JAK2‐V617F mutational burden and is associated with 9p uniparental disomy in myeloproliferative neoplasms
Source: Am J Hematol. 2022 Jan 21;97(4):390–400. doi: 10.1002/ajh.26461 (PMC9306481; doi:10.1002/ajh.26461)
Supplement: Supplementary file 1 — Appendix S1 Supporting Information [file AJH-97-390-s001.docx]

**Supplementary Appendix to**

**PD-L1 Overexpression Correlates with *JAK2*-V617F Mutational Burden and Is Associated with 9p Uniparental Disomy in Myeloproliferative Neoplasms**

Jelena D. Milosevic Feenstra^1^, Roland Jäger^2^, Fiorella Schischlik^3^, Daniel Ivanov^4^,

Gregor Eisenwort^1,4^, Elisa Rumi^5,6^, Michael Schuster^7^, Bettina Gisslinger^4^,

Sigrid Machherndl-Spandl^8^, Peter Bettelheim^8^, Maria-Theresa Krauth^1,4^,

Felix Keil^1,9^, Christoph Bock^7,10^, Mario Cazzola^5,6^, Heinz Gisslinger^4^,

Robert Kralovics^2,7^ and Peter Valent^1,4^

^1^Ludwig Boltzmann Institute for Hematology and Oncology, Medical University of Vienna, Vienna, Austria; ^2^Department of Laboratory Medicine, Medical University of Vienna, Vienna, Austria; ^3^Cancer Data Science Laboratory, Center for Cancer Research, National Cancer Institute, Bethesda, MD, USA; ^4^Department of Internal Medicine I, Division of Hematology and Hemostaseology, Medical University of Vienna, Vienna, Austria; ^5^Department of Molecular Medicine, University of Pavia, Pavia, Italy; ^6^Division of Hematology, Fondazione IRCCS Policlinico San Matteo, Pavia, Italy; ^7^CeMM Research Center for Molecular Medicine of the Austrian Academy of Sciences, Vienna, Austria; ^8^Department of Haematology, Internal Oncology and Stem Cell Transplantation, Ordensklinikum Linz Elisabethinen Hospital, Linz, Austria; ^9^3rd Medical Department, Hematology & Oncology, Hanuschkrankenhaus, Vienna, Austria; ^10^Institute of Artificial Intelligence, Center for Medical Statistics, Informatics, and Intelligent Systems, Medical University of Vienna, Vienna

**Supplemental Methods**

**RNA-sequencing and data analysis**

The dataset underlying this study to evaluate the expression of *PD-L1/2* and other selected genes, has previously been used by Schischlik et al.^1^ In short, RNA isolated from peripheral blood granulocytes of 113 patients with myeloproliferative neoplasms (MPN) and 14 healthy donors was used for preparation of polyA-enriched complementary DNA libraries using various library preparation kits and sequencing was performed on Illumina HiSeq 2000 instrument. Count data was normalized by library size and gene length (FPKM). Further details regarding the sequencing set-up and data analysis have been described in Schischlik *et al*.^1^

**Targeted DNA-sequencing**

DNA samples of 77 patients were analyzed for mutations in 54 genes relevant for myeloid malignancies, using the TruSight Myeloid Sequencing Panel. Samples were processed according to manufacturer’s instructions and equimolar amounts of indexed amplicon-based libraries were pooled and sequenced in 150bp paired-end setting on an Illumina HiSeq3000 instrument. Read alignment and variant calling was performed using the BaseSpace software (Illumina, San Diego, CA) and further data analysis was performed as described previously.^1^

**Microarray analysis**

DNA samples were processed according to the manufacturer’s instructions and hybridized to Genome-Wide Human SNP 6.0 arrays (Affymetrix). The data were analyzed using Genotyping Console version 3.0.2 software (Affymetrix) by applying previously reported criteria for annotation.^2^ All SNP array data reported in this study have previously been published.^3^

**Cell lines and *in vitro* studies**

HEL and SET-2 cells were purchased from Leibniz-Institute DSMZ, German Collection of Microorganism and Cell Cultures. HEL and SET-2 cells were maintained in RPMI 1640 medium, containing 10% or 20% of heat-inactivated FCS, respectively. UT-7 cells were engineered to express various CALR mutants using CRISPR/Cas9 technology as described recently.^4^ All UT-7 sub-clones were grown in Iscove´s Modified Dulbecco´s Medium (IMDM) medium supplemented with 10% FCS. UT-7 parental cells were grown in the presence of 10 ng/ml human TPO, while the *CALR* mutant-transformed UT-7 cell lines were TPO-independent. All cell lines were periodically tested for mycoplasma contamination by conventional PCR using the Venor GeM Classic Mycoplasma Detection Kit (Minerva Biolabs, Berlin, Germany).

**^3^H-thymidine incorporation assay**

To measure the proliferation of *JAK2-* and *CALR-*mutated cell lines ^3^H-thymidine uptake experiments were performed. Cells were incubated in medium containing DMSO or various concentrations of ruxolitinib (1-10000 nM), JQ1 (1-10000 nM) or dBET6 (1-10000 nM) at 37°C for 48 hours. Then, ^3^H-thymidine was added for 16 hours and ^3^H-thymidine uptake was measured as described.^5^ All ^3^H-thymidine uptake experiments were performed in triplicates.

**Supplemental Figures**

**Supplemental Figure 1.**

**Supplemental Figure 1. Diagnosis and disease-driving mutations in the MPN patient cohort (N=113) analyzed by RNA-sequencing.** PMF, primary myelofibrosis; ET, essential thrombocythemia; PV, polycythemia vera; sAML, secondary acute myeloid leukemia

**Supplemental Figure 2.**


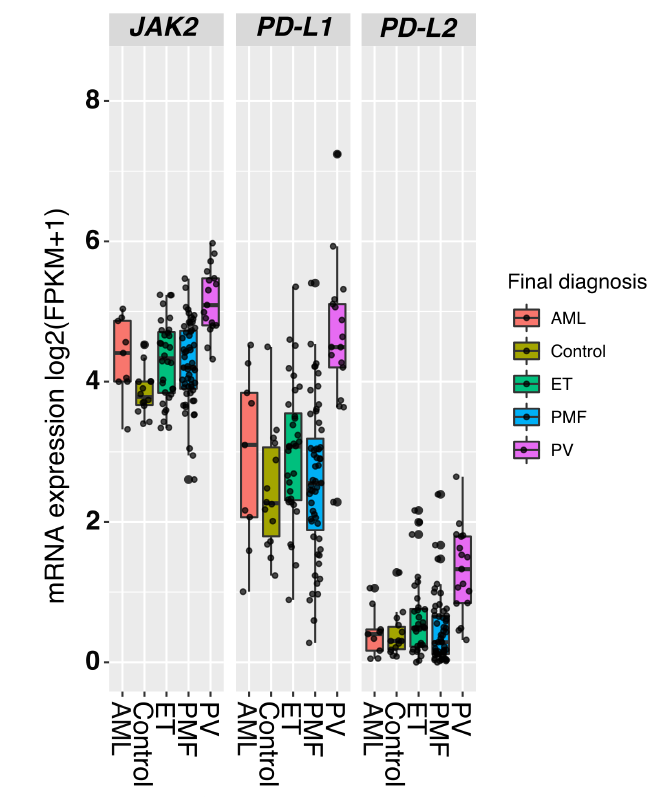


**Supplemental Figure 2. Expression levels of *JAK2*, *PD-L1* and *PD-L2* mRNA detected by RNA-sequencing of granulocytes from 104 MPN patients, 9 post-MPN AML patients and 14 healthy donors.** The box plots show the median (bold horizontal line), interquartile range (box) and total range (whiskers) of mRNA expression levels of *JAK2*, *PD-L1* and *PD-L2* detected by RNA-sequencing of granulocytes from 104 MPN and 9 post-MPN AML patients, as well as 14 healthy donors. PMF, primary myelofibrosis; ET, essential thrombocythemia; PV, polycythemia vera; AML, post-MPN acute myeloid leukemia; Control, healthy donors.

**Supplemental Figure 3.**


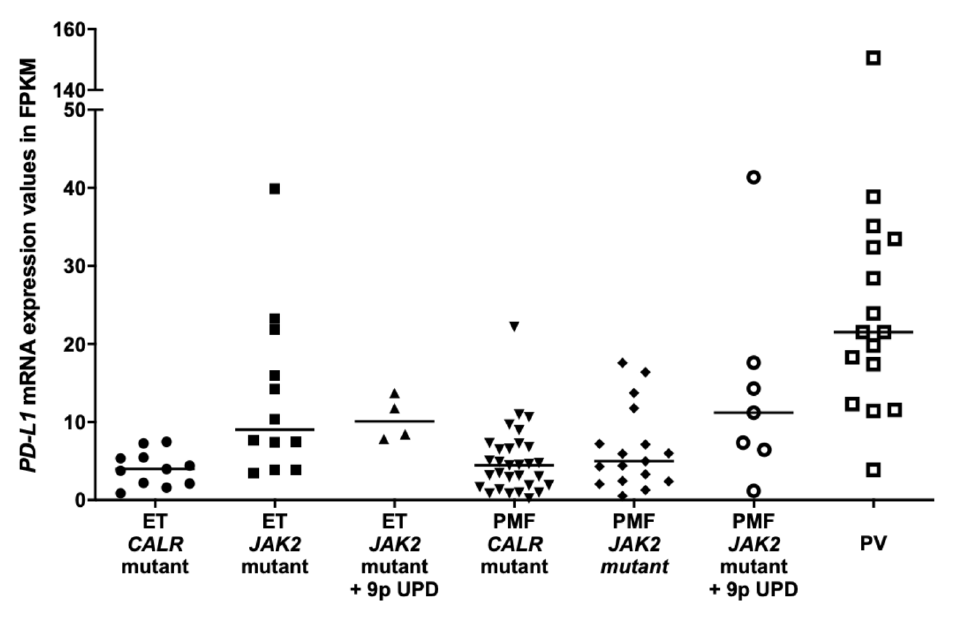


**Supplemental Figure 3. Expression levels of *PD-L1* mRNA detected by RNA-sequencing of granulocytes from 97 MPN patients.** The patient cohort was divided based on diagnosis, driver mutation and presence or absence of chromosome 9p UPD. Only *JAK2*-V617F positive patients for whom data from Genome-Wide Human 6.0 SNP arrays were available were included in this analysis. Median expression levels are depicted as horizontal lines. PMF, primary myelofibrosis; ET, essential thrombocythemia; PV, polycythemia vera; UPD, uniparental disomy.

**Supplemental Figure 4.**

**Relative cell number (%)**

**PD-L1 PE**

**Relative cell number (%)**

**PD-L1 PE**

**PMF**

**Relative cell number (%)**

**PD-L1 PE**

**A**

**B**

**C**

**Supplemental Figure 4. Representative histograms of PD-L1 expression detected on CD34^+^CD45^dim^CD38^−^ cells in one PV (A), one ET (B) and one PMF patient (C).** The isotype-matched control antibody staining is also shown (black open histograms).

**Supplemental Figure 5.**

**Supplemental Figure 5. Surface expression of PD-L1 progenitor cells isolated from bone marrow samples of MPN patients. A)** Using multicolor flow cytometry we detected PD-L1 upregulation on CD34^+^CD45^dim^CD38^+^ progenitor cells isolated from the bone marrow of MPN patients (N=49) when compared to healthy donors (N=7). The horizontal line represents the mean ± standard deviation. **B)** Both *JAK2* and *CALR* mutant MPN patients showed an upregulation of PD-L1 on progenitor cells compared to healthy donors (P<0.001 and P<0.01, respectively). The horizontal line represents the mean ± standard deviation. Mut, mutant; MPN, myeloproliferative neoplasms**Supplemental Figure 6.**

**
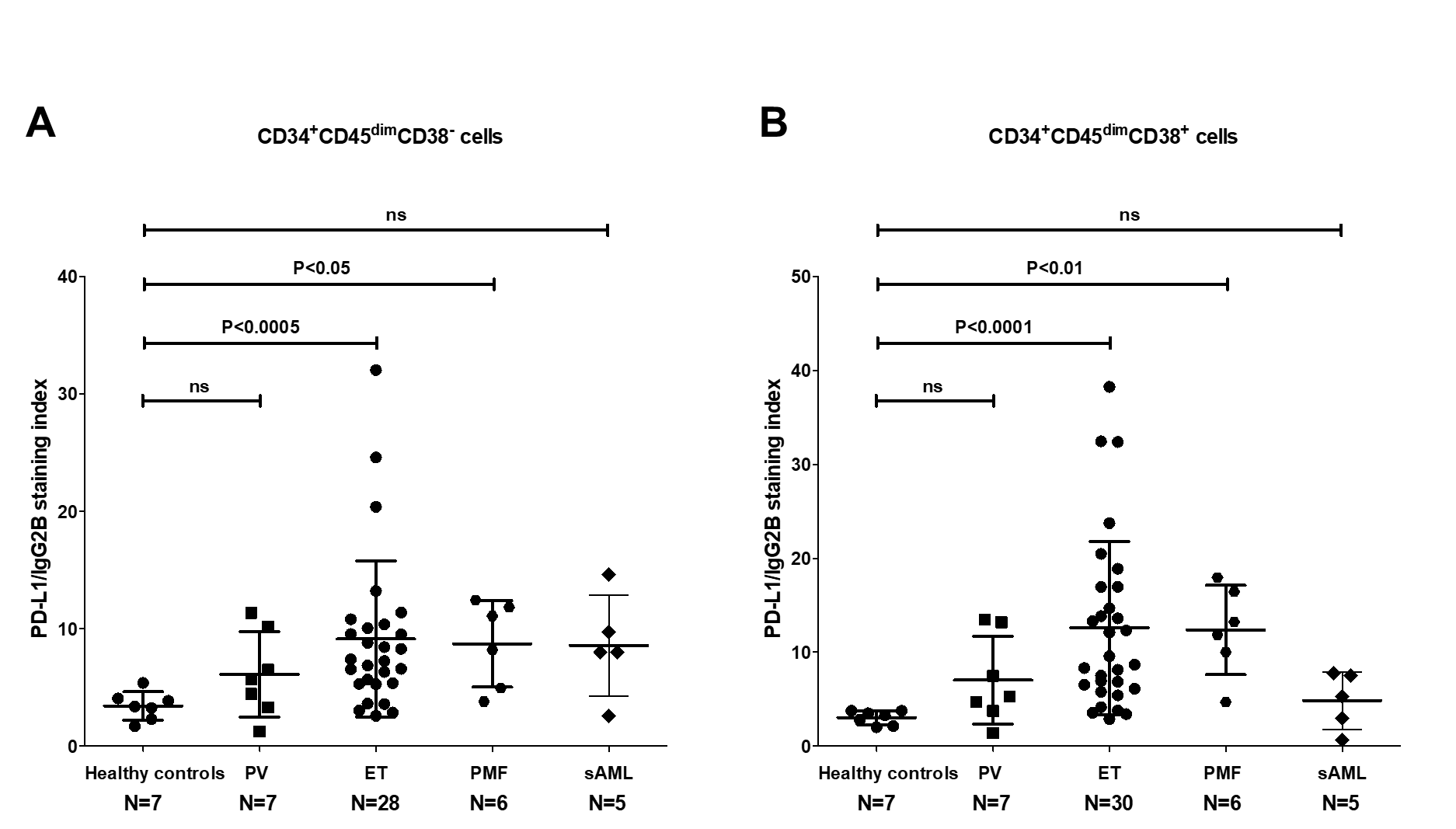
**

**Supplemental Figure 6. Surface expression of PD-L1 on stem and progenitor cells isolated from bone marrow samples of MPN patients. A)** PD-L1 expression was assessed by multicolor flow cytometry on CD34^+^CD45^dim^CD38^−^ putative neoplastic stem cells isolated from the bone marrow of MPN or sAML patients (N=46) or stem cells from healthy donors (N=7). **B)** PD-L1 expression was assessed by multicolor flow cytometry on CD34^+^CD45^dim^CD38^+^ progenitor cells isolated from the bone marrow of MPN or sAML patients (N=48) or healthy donors (N=7). The data in both A) and B) are presented as mean (horizontal line) ± standard deviation. PMF, primary myelofibrosis; ET, essential thrombocythemia; PV, polycythemia vera; sAML, secondary acute myeloid leukemia

**Supplemental Figure 7.**

**Supplemental Figure 7. Lack of surface expression of PD-L2 on stem and progenitor cells isolated from bone marrow samples of MPN patients.** PD-L2 was not expressed on the cell surface of CD34^+^CD45^dim^CD38^−^ cells **(A)** and CD34^+^CD45^dim^CD38^+^ cells **(B)** isolated from the bone marrow of MPN patients or healthy donors as assessed by multicolor flow cytometry. The data horizontal line represents the mean ± standard deviation. **C)** Histogram displaying lack of PD-L2 expression on CD34^+^CD45^dim^CD38^−^ cells in one representative MPN patient. The isotype-matched control antibody staining is also shown (black open histograms).

**Supplemental Figure 8.**

**
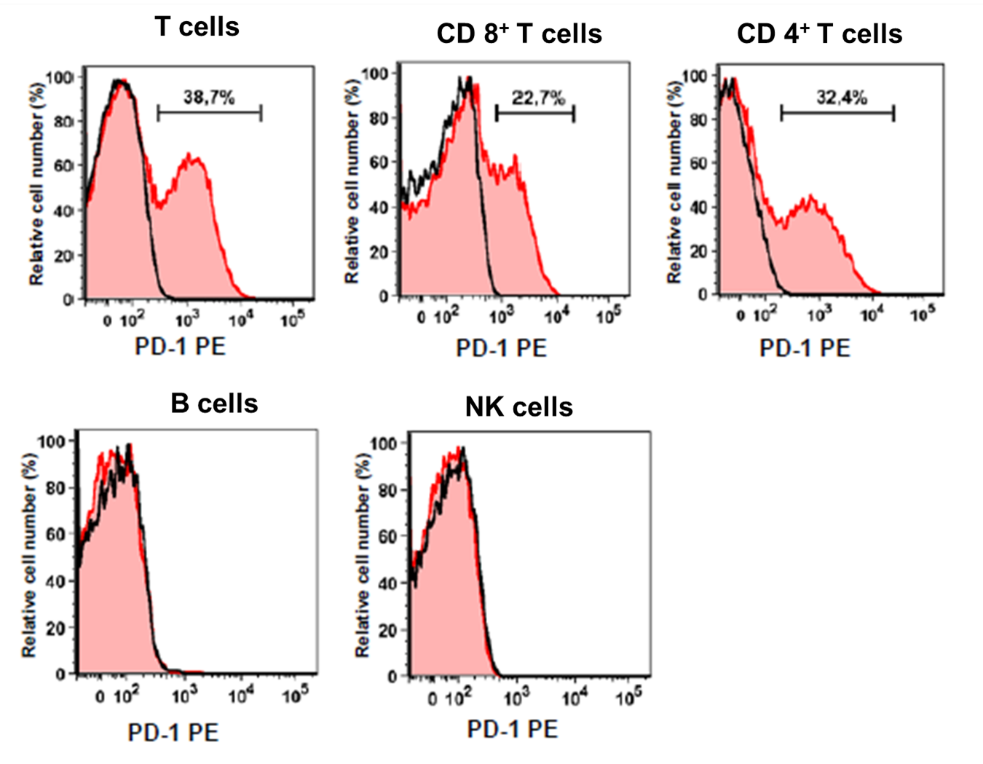
**

**Supplemental Figure 8. Expression of PD-1 on T cells, B cells and NK cells from MPN patients.** The figure shows representative histograms of PD-1 expression on different cell populations detected by multicolor flow cytometry in MPN patients’ fresh bone marrow samples. Isotype control is shown as black lines, while PD-1 is shown as red lines. PD-1 was not found to be expressed in B- and NK-cells of the majority of the samples analyzed.

**Supplemental Figure 9.**

**Supplemental Figure 9. Ruxolitinib, JQ1 and dBET6 downregulate surface expression of PD-L1 on MPN cell lines.** HEL **(A),** UT-7 **(B),** UT-7 CALR del61/wt **(C)** and UT-7 CALR del61/del25 **(D)** cells were incubated with medium or medium containing 200 U/ml of IFN-γ with or without indicated concentrations of ruxolitinib, JQ1 or dBET6 for 24 hours. Drug concentrations were selected at IC20-IC30 for each cell line. Expression of PD-L1 was evaluated using flow cytometry upon 24 hours incubation. The expression of PD-L1 is shown as the staining index which represents the ratio of median fluorescence intensity of PD-L1 and matched isotype control. Results represent expression levels of PD-L1 (shown as staining index) and are expressed as mean ± standard deviation from 3 independent experiments.

**Supplemental Figure 10.**

**
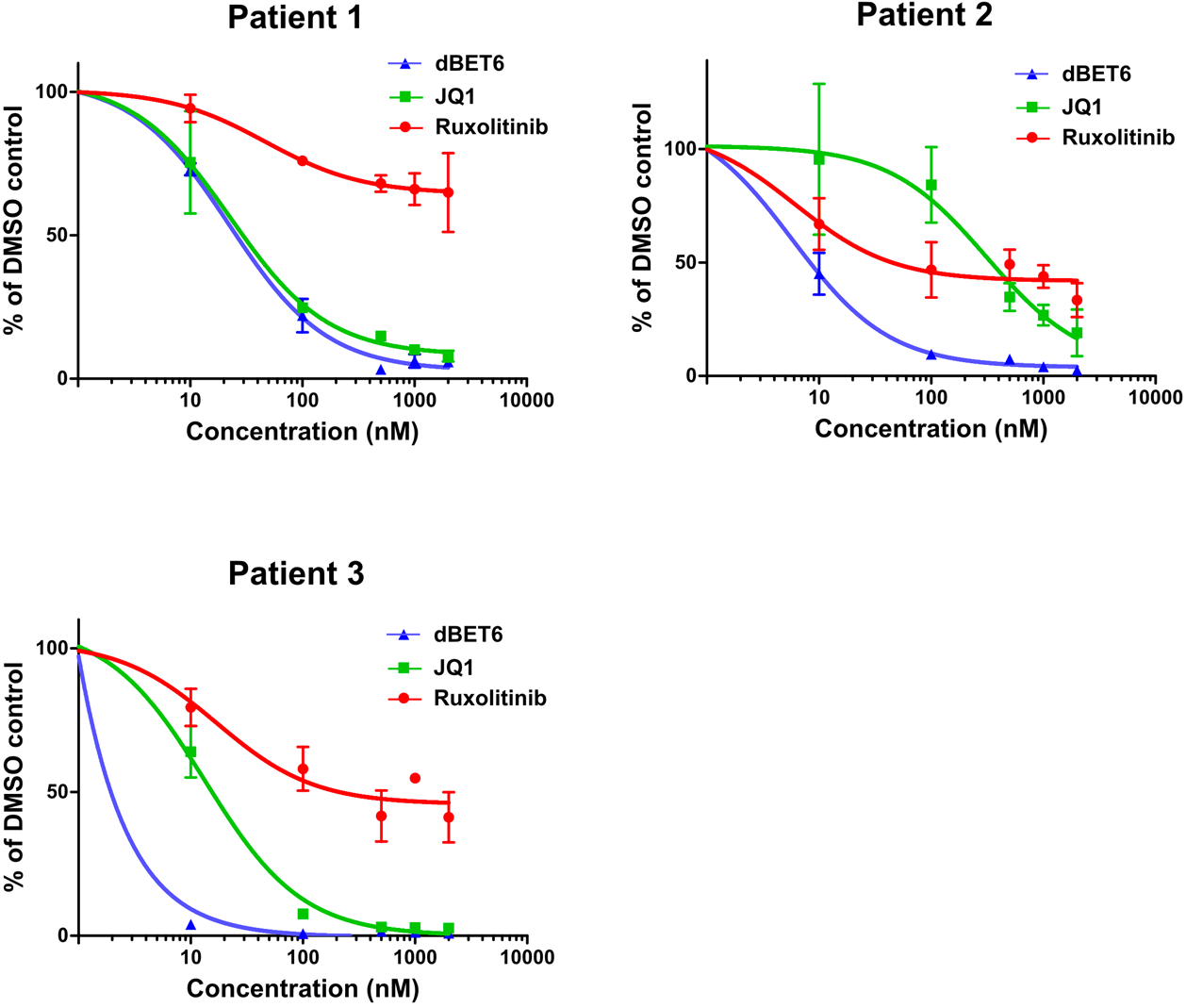
**

**Supplemental Figure 10. Ruxolitinib, JQ1 and dBET6 inhibit proliferation of primary MPN cells.** Primary MPN MNC obtained from two patients with chronic phase MPN (Patient 1 and 2) and one with post-MPN AML (Patient 3) were incubated in medium containing DMSO or various concentrations of ruxolitinib, JQ1 and dBET6 as indicated at 37ºC for 48h. Upon incubation, ^3^H-thymidine was added and 16h later, cells were harvested and bound radioactivity was measured in a β-counter. Results are expressed as percent of control and represent the mean ± standard deviation from triplicates. Patient 1 was diagnosed with PMF with *JAK2*-V617F mutational burden of 56.8% while Patient 2 had ET and *JAK2*-V617F mutational burden of 27%. Patient 3 was diagnosed with post-PV secondary AML with *JAK2*-V617F mutational burden of 72.8%.

**Supplemental Figure 11.**

**Supplemental Figure 11. Ruxolitinib, JQ1 and dBET6 inhibit proliferation of MPN cell lines.** HEL **(A),** SET-2 **(B)**, UT-7 **(C),** UT-7 CALR del58/wt **(D),** UT-7 CALR del61/wt **(E)** and UT-7 CALR del61/del25 **(F)** cells were incubated with medium, DMSO or medium containing various indicated concentrations of ruxolitinib, JQ1 or dBET6 for 48 hours. Cell proliferation was measured in triplicate by uptake of ^3^H-thymidine as described in the methods section. The values were normalized to the DMSO control and represent the mean ± standard deviation from 3 independent experiments.

**Supplemental Tables**

**Supplemental Table 1. Patients’ characteristics**

| **Number of patients** | | **All Patients** | **PV** | **ET** | **PMF** | **MPN-U** | **sAML** |
| --- | --- | --- | --- | --- | --- | --- | --- |
|  |  | 181 | 27 | 64 | 73 | 3 | 14 |
| **Driver mutation**  **(%)** | ***JAK2 mutated*** | 61.6% | 92.6% | 29.7% | 49.3% | 66.7% | 80% |
|  | ***CALR mutated*** | 32.0% | - | 60.9% | 47.9% | 33.3% | - |
|  | ***MPL mutated*** | 1.2% | - | 1.6% | 1.4% | - | - |
|  | **Triple**  **negative** | 4.1% | 7.4% | 7.8% | 1.4% | - | 20% |
| **Gender** | **Male** | 44.8% | 48.1% | 37.5% | 47.9% | 33.3% | 80% |
|  | **Female** | 55.2% | 51.9% | 62.5% | 52.1% | 66.7% | 20% |
| **Median age (years)** | | 59.5 | 64 | 57.5 | 58 | 71 | 52 |
| **WBC, median**  **(range) x10^9^/L** | | 12.6  (1-74.3) | 16.6  (6-74.3) | 12.0  (4.8-21.9) | 12.4  (1.0-31.6) | 6.7  (4.9-8.4) | 9.3  (3.6-19.6) |
| **Hemoglobin, median (range) g/dL** | | 10.4  (3.1-39.3) | 12.7  (8.1-28) | 11.3  (5.4-16.7) | 9.3  (3.1-39.3) | 11.9  (8.8-12.2) | 10.5  (8.2-14) |
| **Platelet count, median (range) x10^9^/L** | | 644.5  (6-2530) | 381  (123-776) | 743.5  (130-1600) | 635  (9-2530) | 945  (77-1239) | 75  (6-1861) |

Abbreviations: PV, polycythemia vera; ET, essential thrombocythemia; PMF, primary myelofibrosis; MPN-U, myeloproliferative neoplasms – unclassifiable; sAML, secondary acute myeloid leukemia; WBC, white blood cell count.

**Supplemental Table 2. Specification of antibodies used in multicolor flow cytometry experiments**

| CD | Antigen | Clone | Conjugate | Species, Isotype | Manufacturer |
| --- | --- | --- | --- | --- | --- |
| n.c. | Isotype control | MOPC-21 | PE | Mouse, IgG1 | BD Biosciences |
| n.c. | Isotype control | 133303 | PE | Mouse, IgG2b | R&D Systems |
| CD3 | TcR | UCHT1 | APC | Mouse, IgG1 | BD Biosciences |
| CD3 | TcR | UCHT1 | APC-Cy7 | Mouse, IgG1 | BD Biosciences |
| CD4 | T4 | SK3 | APC | Mouse, IgG1 | BioLegend |
| CD8 | T8 | SK1 | FITC | Mouse, IgG1 | BD Biosciences |
| CD19 | B4 | 4G7 | FITC | Mouse, IgG1 | BD Biosciences |
| CD34 | HPCA-1 | 581 | FITC | Mouse, IgG1 | BioLegend |
| CD38 | T10 | HIT2 | APC | Mouse, IgG1 | BD Biosciences |
| CD45 | LCA | HI30 | V500 | Mouse, IgG1 | BD Biosciences |
| CD56 | NCAM | MEM-188 | FITC | Mouse, IgG2a | BioLegend |
| CD273 | PD-L2 | MIH18 | PE | Mouse, IgG1 | BioLegend |
| CD274 | PD-L1 | 29E.2A.3 | PE | Mouse, IgG2b | BioLegend |
| CD279 | PD-1 | EH1.2H7 | PE | Mouse, IgG1 | BioLegend |
|  |  |  |  |  |  |

Abbreviations: CD, cluster of differentiation; n.c., not clustered; PE, phycoerythrin; FITC, fluorescein isothiocyanate; APC, allophycocyanin; Ig, immunoglobulin; TcR, T cell receptor; LPSR, lipopolysaccharide-related antigen; FcγRIIIa, Fc receptor gamma IIIa; HPCA-1, human precursor cell antigen-1; LCA, leukocyte common antigen; NCAM, neural cell adhesion molecule; ENPP3, Ectonucleotide Pyrophosphatase/Phosphodiesterase 3; PD-L1/2, Programmed death-ligand 1/2; PD-1, Programmed cell death protein 1.

Company Locations: BD Bioscience, San José, CA, USA; R&D Systems, Minneapolis, MN, USA; Dako, Glostrup, Denmark; Miltenyi Biotec, Bergisch Gladbach, Germany; BioLegend, San Diego, CA, USA; Santa Cruz Biotechnology, Dallas, TX, USA.

**Supplemental Table 3. Observed frequencies of all possible haplotypes spanning both *JAK2* and *PD-L1* in an MPN cohort (N=272) and a population-matched non-MPN control cohort (N=1620). Calls homozygous for both loci were further used for haplotype-based association analysis (Table 2).**

| Genotypes rs10974944_rs4143815 | N (MPN) | N (control) | % MPN | % control | Homozygous genotypes |
| --- | --- | --- | --- | --- | --- |
| CC_GG | 35 | 425 | 13% | 26% | *JAK2*_major, *PD-L1*_major |
| CC_GC | 31 | 343 | 11% | 21% | n.a. |
| GC_GC | 69 | 323 | 25% | 20% | n.a. |
| GC_GG | 41 | 271 | 15% | 17% | n.a. |
| GG_GC | 23 | 73 | 8% | 5% | n.a. |
| GC_CC | 13 | 64 | 5% | 4% | n.a. |
| CC_CC | 11 | 62 | 4% | 4% | *JAK2*_major, *PD-L1*_minor |
| GG_GG | 27 | 45 | 10% | 3% | *JAK2*_minor, *PD-L1*_major |
| GG_CC | 22 | 14 | 8% | 1% | *JAK2*_minor, *PD-L1*_minor |

n.a., not applicable; MPN, myeloproliferative neoplasms.

**Supplemental References**

1. Schischlik F, Jager R, Rosebrock F, et al. Mutational landscape of the transcriptome offers putative targets for immunotherapy of myeloproliferative neoplasms. *Blood*. 2019;134(2):199-210.

2. Milosevic Feenstra JD, Nivarthi H, Gisslinger H, et al. Whole-exome sequencing identifies novel MPL and JAK2 mutations in triple-negative myeloproliferative neoplasms. *Blood*. 2016;127(3):325-332.

3. Klampfl T, Harutyunyan A, Berg T, et al. Genome integrity of myeloproliferative neoplasms in chronic phase and during disease progression. *Blood*. 2011;118(1):167-176.

4. Jia R, Balligand T, Atamanyuk V, et al. Hematoxylin binds to mutant calreticulin and disrupts its abnormal interaction with thrombopoietin receptor. *Blood*. 2020.

5. Hadzijusufovic E, Keller A, Berger D, et al. STAT5 is Expressed in CD34(+)/CD38(-) Stem Cells and Serves as a Potential Molecular Target in Ph-Negative Myeloproliferative Neoplasms. *Cancers (Basel)*. 2020;12(4).
